# How Screen Time Affects Greek Schoolchildren’s Eating Habits and Functional Food Consumption?—A Cross-Sectional Study

**DOI:** 10.3390/nu17081311

**Published:** 2025-04-09

**Authors:** Irene Chrysovalantou Votsi, Antonios Ε. Koutelidakis

**Affiliations:** Laboratory of Nutrition and Public Health, Department of Food Science and Nutrition, University of the Aegean, Leoforos Dimokratias 66, 81400 Myrina, Greece; fnsd20004@fns.aegean.gr

**Keywords:** screen time, functional foods, KidMed, children, primary school, schoolchildren, eating habits, parents

## Abstract

Background: Television (TV), video games, PC and devices such as tablets and smart phones have become part of everyday life at an ever-younger age. Increased screen time correlates with unhealthy eating habits among children. Methods: 374 children aged 9–12 years and their parents (n = 159), from 3 schools in Lemnos and 5 schools in Thessaloniki, Greece, took part in this cross-sectional study. The children completed the KIDMED score and a questionnaire about their physical activity, time spent watching TV, PC and playing electronic games, the frequency of cooking or shopping with their parents, the frequency of eating fast food, soft drinks and Functional Foods (FFs). Statistical analysis was performed with SPSS-29.0, using One Way ANOVA and Pearson chi-square. Results: As the hours of TV viewing increased, so did the percentage of children who consumed soft drinks (*p* = 0.03). A statistically significant association detected between television (*p* = 0.024), video games (*p* = 0.028), all screen categories (*p* = 0.011) and fast-food consumption. Increased screen time is associated with a higher weekly consumption of fast food (*p* = 0.011). The more hours children spent in front of screens, the less adherence they had to the Mediterranean Diet (*p* = 0.001) and less natural FFs consumption (*p* = 0.001). Conclusions: The results suggest that screen time seems to affect children’s eating behaviors. The study concluded that the longer the screen time, the unhealthier the dietary habits of schoolchildren become. Future research should focus on reducing screen time, as a means of improving dietary patterns and potentially reducing childhood obesity.

## 1. Introduction

Screen time (ST) is becoming an important factor influencing dietary habits in children. It encompasses the duration spent using screens, including computers, televisions, video games, smartphones and tablets. Excessive screen time has been linked to various health concerns in children, such as body weight issues, reduced sleep duration and mental health problems [1]. Numerous cross-sectional and prospective studies indicate a strong correlation between unhealthy eating habits and television watching among children. Specifically, greater television viewing time is associated with higher consumption of sugary drinks and non-whole grain products [2]. Furthermore, watching TV is linked to poorer dietary choices and lower intake of fruits and vegetables among children [3].

Moreover, the rise in sedentary behaviors, such as ST and lack of physical activity, is connected to unhealthy eating patterns in European adolescents. In particular, there is evidence of a shift from the traditional Mediterranean Diet (MD) towards a preference for energy-dense foods typical of Western diets, particularly in Mediterranean regions [4]. Recent research illustratesthat high consumption of sugary soft drinks and low nutrient dense food like fast food can have significant health consequences for children and adolescents, including obesity, Type 2 Diabetes, reduced bone density, dental problems, mental health difficulties and nutritional deficiencies [5,6,7,8,9,10,11,12,13,14,15,16].

While engaging in high-quality screen activities can fulfill certain educational and entertainment needs, excessive exposure to screens may negatively impact children’s physical health, cognitive abilities and psychosocial development. Consequently, managing screen time during childhood and adolescence is essential, balancing the associated risks and benefits [17]. The American Academy of Pediatrics (AAP) advises that children under 18 to 24 months should completely avoid screen media, while children older than this age should limit their screen time to no more than one hour per day [17,18]. Similarly, the World Health Organization (WHO) states that infants in their first year should not be exposed to digital screens. For children aged 2 to 5 years, screen time should be restricted to a maximum of one hour daily [17,19]. The Canadian Pediatric Society has issued similar guidelines, recommending that children younger than 2 years avoid screens entirely and that those aged 2 to 5 limit their screen time to less than one hour per day [20]. There is growing concern regarding the effects of screen time, particularly on mobile phones, on the health and well-being of children and adolescents.

The environment in which a child is raised plays a crucial role in shaping their healthy lifestyle choices, withtwo major influences being the family and the school [21,22]. The family, including parents and caregivers, has a significant influence as they can instill beliefs and attitudes that foster a positive perspective on healthy nutrition and eating habits while also expressing essential cultural and emotional connections to food [22,23]. The Mediterranean lifestyle is beyond a healthy mainly plant-based diet characterized by culture, human contact, moderation and physical activity and has been acknowledged as the most appropriate shield mainly for obesity-related disease prevention, and the roadmap to longevity, wellbeing, and health care sustainability. Several studies in children and adolescents have found an inverse correlation between the degree of adherence to Mediterranean diet and BMI (Body Maas Index) [24]. Additionally, multiple studies have found that parental BMI is associated with that of their children [25].

Although many aspects of diet and lifestyle influence metabolic status and disease progression throughout life, emerging evidence suggests that the frequency and timing of meals also play a significant role in overall health [26,27]. In this context, Functional Foods—those with specific health benefits beyond basic nutrition—are increasingly recognized for their role in maintaining a healthy lifestyle and reducing the risk of various diseases. Most foods, including fruits, vegetables, cereals, meat, fish and dairy, contain functional ingredients which is responsible for improving the health [28].

The present study aimed to investigate (1). The connection among screen time, dietary habits and Functional Food consumption in children aged 9 to 11 years. Research focusing on this age group is particularly crucial, as lifestyle behaviors, such as poor diet, are changeable and often formed during childhood or young adulthood. The transition from childhood to adolescence brings about various stressors that can significantly affect individuals’ health-related lifestyle decisions. (2). At the same time, we decided to create family clusters and correlate them with screen time, physical activity and fast food consumption. The variables that we selected to create this clusters were BMI scores, number of meals per day, frequency of Functional Foods’ consumption and MedDiet and KidMed score, because of their well-established associations between adherence to the Mediterranean diet and children’s health outcomes [29].

Additionally, it is important to emphasize that a variety of studies in the literature have explored the impact of screen time on eating habits; however, this is the first study to specifically examine the consumption of Functional Foods within the context of those eating habits.

## 2. Materials and Methods

### 2.1. Participants and Study Procedures

This is a cross-sectional study using a sample which was composed of Greek schoolchildren aged between 9–11 years old (n = 373) and their parents (n = 159). Data was collected between May 2022 and July 2022. Children and their parents/guardians (hereon referred to as “parents”) were recruited from 8 elementary schools located in Thessaloniki and Lemnos. To include students from different city areas (north, south, east, west and central), one school per region was randomly selected. 15 primary public schools in the town (10 schools) and on the island (5 schools) were invited to participate in the study and only 8 of those agreed to participate (5 schools from Thessaloniki and 3 schools from Lemnos). Participating in public schools were equally spread over the area of Thessaloniki and Lemnos. The parents of 70% of children gave written informed consent and only these children participated in the research (373 pupils from 538). Informed consent was obtained from parents two weeks before the data collection day. We visited schools where children’s eating/physical habits and watching screen time were investigated using printed questionnaires. After informing the children about the purpose of our visit, they began to complete their questionnaires. Questionnaires were self-reported by the students at school in the presence of a dietitian researcher who was available to answer any possible questions. The participants completed a diet and lifestyle questionnaire, which included a self-reported measure of screen viewing and a food frequency questionnaire (FFQ). We didn’t take any anthropometric measurements (weight or height) of children; we relied on the weight and height that the parents themselves reported to us about their children in their electronic questionnaires. The parents were invited to answer a similar questionnaire with their kids at the same period, electronically, via the Google Forms platform and we asked to be completed by one parent of each child.

#### 2.1.1. Inclusion and Exclusion Criteria

The Inclusion criteria included: (1) students aged 9–11 years; (2) students enrolled in public elementary schools; (3) students who had returned the informed consent form authorizing their participation, signed by their parents or guardians.

Exclusion criteria were: (1) students with a physical disability or having a limitation for doing any activity usually carried out by children due to any health issue, (2) students who had health problems and needed to follow a special dietary pattern that excludes certain foods from their diet or who had been diagnosed with a food disorder such as anorexia nervosa, bulimia nervosa, dysphagia etc., (3)students and parents who didn’t speak Greek with sufficient fluency were excluded from the study to avoid errors in data collection responses.

#### 2.1.2. Ethical Approval

This study was conducted according to the guidelines laid down in the Declaration of Helsinki of 1975 and all procedures involving research study participants were approved by the Ethical Review Board of the Ministry of Education and Religious Affairs and the Ethical Committee of University of the Aegean, approved the study before its commencement (approval No. 22/13 February 2022).

### 2.2. Measures

Parents’ self-reported body weight and height data were used to calculate their Body Mass Index (BMI). Parents’ weight status was assessed using BMI, calculated using the formula: weight (kg)/height (m^2^). The weight status variable was classified into four categories: Underweight, Healthy Weight, Overweight and Obesity [30]. The BMI-for-age cut-off points of the WHO child growth standards were used for children [31].

### 2.3. Demographic and Socioeconomic Characteristics

Sociodemographic information about parents including age, gender, smoking and socioeconomic status (SES) was collected. SES was assessed through the following questions: educational level, type of profession, income and number of family members. Parental educational level was classified as low (lower general secondary education, lower vocational training and primary school or less), medium (intermediate vocational training, higher general secondary training and pre-university education) or high (completed higher vocational training and University) based on the highest completed education level of both parents. Type of profession was classified as private or public employee, freelancer, self-employed, domestic worker and farmer. Income was classified as low, medium and high.

### 2.4. Dietary Assessment

Children’s questionnaire includes questions about how many meals consume every day, the frequency of breakfast and school snacks consumption and the frequency of fast foods Functional Foods and beverages consumption. To evaluate adherence to the Mediterranean Diet by the students the KIDMED score was used. The KIDMED score classify participants into three categories: low adherence (3 or fewer points), medium adherence (4–7 points) and high adherence (8 or more points) [32].

To evaluate adherence to the Mediterranean Diet by the parents, the MedDiet Score was used. In particular, for the consumption of food items that are close to the Mediterranean diet scores 0 for rare or no consumption, to 5 for almost daily consumption, were assigned, whereas, for the consumption of foods that are away from this traditional diet (like meat and meat products), the opposite scores were assigned (i.e., 0 for almost daily consumption to 5 for rare or no consumption). For alcohol consumption, score 5 for the consumption of less than 3 wine glasses per day and, progressively, score 0 for the consumption of more than 7 wine glasses per day. Thus, the range of the diet score is between 0 and 55 [33].

### 2.5. Functional Foods

Participants reported the frequency of consumption of Functional Food (FF) items based on daily, weekly and monthly intakes over the past year. A FFFQ (Functional Food Frequency Questionnaire) with 50 food items (more natural and some enriched Functional Foods) was given to the subjects using the following categories of frequency consumption: never, rarely, two to three times a month, once to two times a week, three to five times a week, daily. Functional Foods were selected according to the position paper of Papagianni et al. who developed and validated a new FFFQ which includes 48 food groups and 28 individual foods (food subgroups), for a total of 76 food groups, which were categorized mainly based on the major food groups they belong to, but also on their bioactive component [34]. We decided to select these 50 Functional Foods to be included in the questionnaire, thinking that 1. They are more widespread and more frequently consumed by children and parents than other Functional Foods we had on our list, and 2. That we were limited in time and couldn’t include more than 50 FFs in the questionnaire for the participants to answer. Some examples of natural and enriched Functional Foods used in the FFFQ were: citrus fruits, strawberries, blackberries, pomegranate, cauliflower, broccoli, spinach, beans, walnuts, olive oil, salmon, eggs, dairy, fortified juices, breakfast cereals and oat cereal bars.

### 2.6. Physical Activity

Parents were asked to indicate the average number of days per week and weekends that they spent time on physical activity (none, 1–2 times/week, 3–4 times/week, >4 times/week).

### 2.7. Screen Time

To assess children’s TV watching time and screen time, pupils responded to the question “On average how many hours per day do you watch TV’ and ‘How many hours per day do you spend in front of screens. Screen as detected as playing on the computer, playing video games, using tablet or/and a mobile phone. Response options were 1 = none; 2 = 1–2 h a day; 3 = 2–3 h a day; 4 = 3–4 h a day; 5 = >4 h a day.

To consider increased use of screen devices, the recommendation of the American Academy of Pediatrics was used, which considers more than two hours/day for children over 2 years old as excessive use [35]. Pupils who reported use lower than the cutoff point they were considered as low/normal screen use.

### 2.8. Statistical Analysis

Statistical analyses were performed using IBM SPSS for Windows, version 29.0. Differences in characteristics between boys and girls were studied by chi-squared tests of association and between fathers and mothers, by chi-squared tests of association and independent samples *t*-tests. Associations between variables related to screen time (TV viewing, tables, videogaming etc.), eating habits and BMI, MedDiet Score or Kidmed were explored with chi-squared tests of association and One-Way Analysis of Variance (ANOVA) with Bonferroni corrections, respectively. Two-Step Cluster Analysis was utilized to explore family segments relative to dietary habits (BMI of parents and children, MedDiet score, Kidmed score, parents’ consumption of functional food, children’s consumption of natural functional food, and children’s number of meals per day). The associations of the extracted family clusters with demographic, dietary variables and screen time were explored by chi-square tests. Normality of continuous variables was assessed by Kolmogorov-Smirnov tests. The statistical significance level was set at an alpha value of <0.05.

## 3. Results

This study included 373 schoolchildren (162 girls and 211 boys) aged 9 to 11 years, of 4th, 5th and 6th grade of primary school. The study was conducted in two areas, in Thessaloniki (city) and in Lemnos (island-province) in eight schools from different regions. Five schools from Thessaloniki and three schools from Lemnos were randomly selected to participate. Schoolchildren’s descriptive characteristics are shown in Table 1.

A total of 138 (38.8%) schoolchildren spent 1–2 h of screen time per day and 91 (25.6%) schoolchildren spent ≥3 h of screen time per day. Half of the children (54.4%) watch television for 1 h per day. Moreover, 118 (38.7%) children played video games 2–3 h per day. It was noticed that girls spend more hours in front of screens, watching TV and playing video games, compared to boys (Table 1).

As for their dietary habits, 204 (71.1%) pupils, slightly more than half of the sample, consume breakfast every day before leaving home. 242 from 373 (65.8%) children consumed soft drinks. KIDMED index results showed us that 33 (12.4%) pupils had very low diet quality, 130 (48.9%) pupils had average Mediterranean Diet adherence, improvement needed to adjust intake to Mediterranean patterns and 103 (38.7%) had good adherence to the principles of the Mediterranean Diet (Table 1).

### 3.1. Children’s Screen Time and Eating Habits

Further data analysis was applied to estimate the effect of screen time categories on specific dietary habits (Table 2). Specifically, chi-square associations showed that skipping breakfast, consuming fast food and soft drinks frequently, were associated with increased screen time. In detail, comparisons between all categories of screen time showed that the more time children spend in front of screens, the greater the likelihood of developing unhealthy dietary habits.

Cooking with parents and the hours that they spent playing video games was found to be statistically significant (*p* = 0.035). The more hours the children cooking with their parents, the less they play video games. In addition, it was found statistically significant association between frequency of taking homemade snacks to school and hours in front of screens (*p* = 0.026). The more hours the children spent in front of screens, the less often they took homemade snacks to school. Also, there is a statistically significant association between hours in front of screens and daily breakfast consumption (*p* = 0.010). Specifically, as the amount of time children spend in front of screens increases; the percentage of those who eat breakfast daily decreases. Further, it was found that there was a statistically significant association between soft drinks consumption and hours of television viewing (*p* = 0.03). As the hours of TV viewing increased, so did the percentage of children who consumed soft drinks (from 50% increased to 88.5%). It was noticed the same with the hours that pupils spent in front of screens and the soft drinks consumption (*p* = 0.001). Students who are in front of screens for >3 h consume soft drinks by 77%.

Regarding fast food consumption, we found a statistically significant association with television (*p* = 0.024), video games (*p* = 0.028) and all screen categories (*p* = 0.011). Longer periods of screen time are often associated with more frequent consumption of fast food each week. Also, it was found the same results in video games.

Table 3 provides that screen time have a statistically significant association with MD adherence (*p* = 0.001) and natural FFs consumption (*p* = 0.001). The more hours children spent in front of screens, the less they adhered to the Mediterranean Diet and the lower their consumption of natural Functional Foods. A statistically significant association is shown in those children who watch TV every day; they have lower KidMed score than those children who watch TV 1-2 times/week. Finally, the more hours they played with video games the lower the adherence they had to MD (*p* = 0.013). The same thing it was observed with natural FFs (*p* = 0.019).

### 3.2. Family Clusters (Parent-Child Dyads)

There were 159 parents (139 mothers and 20 fathers) who participated in the study. Participant demographic information is available in Table 4.

The results of parents’ BMI, parents’ MedDiet Score and children’s BMI total and by gender are in Table 5.

A statistically significant correlation of children’s BMI (Body Mass Index) with KidMed score was found. A higher KidMed score was associated with a lower BMI in children. This indicates that better adherence to the principles of the Mediterranean Diet corresponded to a lower Body Mass Index.In addition, children’s BMI is positively related to parents’ BMI. An increase in parents’ BMI was linked to a rise in children’s BMI. (Table 6).

We chose some variables that are important for the parents’ health and some variables that are important for the child’s health and made specific family profiles (FAMILY CLUSTERS). We put determinant variables in the analysis clusters. We created the 2 groups and correlated them with the screens, physical activity andfast food consumption. Clusters were produced with the Two-Step Cluster analysis method and the solution’s silhouette score was 0.2, indicating a fair cluster quality. Input variables in the cluster analysis were children and parents’ BMI scores, MedDiet and KidMed scores, number of meals per day, children and parents’ frequency of functional foods’ consumption.

The cluster analysis showed that our sample was divided into 2 groups. We had a total of 137 parent-child groups. The 1st group consisted of 70 parent-child dyads (51.1%) and the 2nd group consisted of 67 parent-child dyads (48.9%). The families which belong to the 1st cluster/1st group had higher adherence to MD (both parents and children) and this is associated with lower BMI (both parents and children), higher Functional Foods consumption and more meals of the children per day. The 2nd cluster/2nd group had families with lower adherence to MD, higher BMI, lower Functional Foods consumption and a lower number of meals that the children eat per day (Table 7).

Cluster analysis showed that regarding fast food, children have a worse diet than their parents. We observed no significant association in fast food between parents, but there is between children (*p* = 0.049). In families that adopt a healthier diet in terms of MedDiet score, BMI, FFs consumption, their children’s fast-food consumption is lower. Soft drinks and cluster weren’t found to have association (Table 8).

## 4. Discussion

It was found that there were statistically significant association between soft drinks consumption and hours of television viewing, playing video games and hours that pupils spent in front of different categories of screens. As the hours of watching increased, so did the percentage of children who consumed soft drinks. Similar findings observed in a Spanish cross-sectional study, which found that high levels of screen time were associated with a greater frequency of beverage consumption [36]. In a Swedish sample of 2–9 years old children there were associations between screen habits and sweetened beverage consumption [37]. Moreover, in the cross-sectional ToyBox study, which was conducted in several European countries (Belgium, Bulgaria, Germany, Greece, Hungary, Italy, Netherlands, Poland, Spain), it was shown that children who spend more time on screens tend to consume more sugary beverages. [38]. Identical conclusions reached from cross—sectional survey of Kanyinga and their colleagues, whose target group were adolescents between the ages of 10 and 19 years old. There were inversely associations with consumption of sugar-sweetened beverages (SSBs) and energy drinks (EDs) [39]. Kenney et al. found that TV viewing and other screen device use, including use of smartphones and tablets, increase intake of SSBs, independently of one another [22] as confirmed in our study. Also, screen time was positively associated with soft drink consumption in Huo’s and Falbe’s studies [1,40]. Hueso and their colleagues found that Spanish children who spend more time on screens are more likely to consume unhealthy snacks and sugary beverages [41]. One possible reason for these results could be that children who spend more time in front of screens are more likely to passively consume foods high in fats, salt and sugar. This is because they tend to pay less attention to their eating habits while engaged in other activities. Additionally, many advertisements shown during children’s television (or online) programming promote foods and beverages that are high in calories, salt, fat and sugar [36].

A statistically significant correlation of children’s BMI (Body Mass Index) with KidMed score was found. A strong, inverse association was observed between KIDMED score and children’s weight status in a recent cross—sectional study in Greece [31]. Previous findings by Kanellopoulou et al. (2021) among Greek children revealed similar values of the KIDMED index and children’s BMI [42]. Furthermore, a recent Greek study among children aged 6–18 years revealed that participants who are overweight/ obese had higher proportions of unhealthy dietary habits than those with normal weight [43]. In line with our findings in a current study that included students from ten geographical areas of Greece, showed that overweight and obese children had lower KIDMED scores compared to normal-weight children [44]. In contrast to our results, new study on Italian children (mean age 11 ± 3.4 years), they didn’t find any association between KIDMED scores and BMI [45]. Also, in our research children’s ΒΜΙ was not found to be associated with screen time, but in a cross- sectional study in Germany found that higher screen time was associated with a higher likelihood of being overweight or obese among adolescents [46]. In Robinson’s survey found a significant association between increased screen media exposure (such as television, video games and computers) and higher rates of obesity among American children and adolescents [47]. It was observed a similar trend in other recent studies, which indicated that greater screen time was linked to higher BMI [48,49,50,51,52].

Regarding fast food consumption, we found a statistically significant association with all kinds of screens (television, video games, tablets, laptops, computers, mobile phones). Increased ST is associated with a higher weekly frequency of fast-food consumption. Likewise, these results are in line with a few previous studies that have examined similar hypotheses. In a Spanish Cross-Sectional Study with children 1–14 years old, is found that increased leisure ST is often associated with higher intake of junk food. Children who spend more time on screens may be more likely to consume unhealthy snacks and sugary beverages [53]. Consistently with a study from WHO European Childhood Obesity Surveillance Initiative, screen time was found to be associated with consumption frequencies of energy-dense, micronutrient-poor foods which underline the importance of limiting children’s screen time exposure [54]. Finally, in a review conducted a few years ago found evidence that the time spent watching television, playing on the computer and total screen time were inversely related to diet quality among children. The higher consumption of a potentially cariogenic diet was found among children with excessive screen time [55].

Our results show that ST has a statistically significant association with MD adherence. The more hours children spent in front of screens, the less adherence they had to the MD. Similar to our results, PASOS study revealed that greater amount of screen time was associated with worse adherence to the MD [23]. In agreement with our findings, the HELENA study investigated that screen time and MD adherence are inversely associated in European adolescents [4]. Further, in a current study investigated the relationship between the use of different types of popular media among 10- to 11-year-old schoolchildren and their commitment to the MD. The findings revealed that engaging in electronic gaming and watching television or streaming content is significantly linked to a reduced adherence to MD [56].

Additional, it was found a statistically significant correlation between ST and FFs. The more hours children spent in front of screens, the less natural FFs consumption had. Due to the fact that no research was found correlated the consumption of Functional Foods with ST, used studies examining fruits and vegetables (which considered natural FFs) is referenced to discuss this finding. In a recent observational study, among adolescents, watching television has been linked to reduced consumption of vegetables, calcium-rich foods, and whole grains [57]. Similarly, several studies have shown that ST has a negative impact on vegetable and fruit consumption [1,58,59,60].

It was noticed that girls on our sample spend more hours in front of screens, watching TV and playing video games, compared to boys. Ιn contrast to research by Ryciak et al. which observed that the average ST was higher among boys [61]. Research conducted by Ruzic-baf and her colleagues also revealed that boys engage in gaming more than girls [62]. The literature indicates that male gamers tend to begin playing video games at a younger age, engage in gaming more often, and dedicate more time to playing compared to female gamers [63] A recent survey from the Norwegian Media Authority shows that between 2018 and 2020, the number of girls aged 9–18 who regularly play video games increased. By 2020, 96% of boys and 76% of girls reported engaging in regular gaming, compared to 96% of boys and 63% of girls in 2018 [64]. Female video game players have significantly increased in the past few years [65]. While many studies indicate that gender is an independent factor linked to excessive screen time, there has been limited research on gender differences regarding its prevalence, associated factors, and the effects of screen time among children [66].

The cluster analysis showed that the families which belong to the 1st group had higher adherence to MD (both parents and children) and this is associated with lower BMI (both parents and children), higher Functional Foods consumption and more meals of the children per day than the 2nd group. Our findings align with previous research that also demonstrated the positive impact of parents’ healthy eating behaviors which effectively improve their children’s diet and weight status [67,68,69].

Our study has several limitations. First, we didn’t check the hours that the children’s parents spend in front of screens (parental screen habits). Second, we didn’t measure separately the screen time that kids spent on weekdays and/or weekends. Other limitation, was that the number of our sample was not representative of the primary schools that there are in the area of Thessaloniki and Lemnos. In addition, limitations included the limits of a cross-sectional design only including one time-point, the fact that there could be recall bias in the study, and the possibility for social desirability bias, and potential confounders that were not controlled (e.g., parental screen habits, psychological stress). Several of the above limitations were attempted to be reduced through education and the appropriate scientific role of the researchers.

## 5. Conclusions

Conclusively, increased screen time correlates with poorer dietary habits and lower Functional Food consumption among schoolchildren. There is a positive relationship between screen time and unhealthy eating behaviors. Consequently, the education system should focus on encouraging schoolchildren to participate in physical activities, limit their screen time and consume healthier foods like fruits and vegetables. Intervention programs should focus on setting guidelines for classroom screen use, balancing educational use with physical and social activities, teaching responsible screen use, and incorporating more interactive, hands-on learning to reduce screen reliance.

Parents also play a key role in managing screen time and feeding behaviors. They should set clear screen time rules based on their child’s age, model balanced screen use and encourage outdoor play, sports, reading and other non-screen activities. Policymakers should launch campaigns to educate families about the risks of excessive screen time, collaborate with health organizations to create national screen time guidelines and support schools and families in promoting physical activity and limiting screen use. Future research could focus on evaluating the effectiveness of intervention programs that reduce screen time while promoting healthy eating and functional food consumption, studying the impact of digital marketing on children’s food choices, especially Functional Foods, and how screen time influences this and exploring app-based interventions that use gamification and educational content to encourage healthy eating and limit screen time.

## Figures and Tables

**Table 1:** Descriptive characteristics of children, total and by gender.

Variables		Gender						Chi-Square Test of Association *p*-Value
		Total		Girl		Boy		
		N	%	N	%	N	%	
**Region**	City (Thessaloniki)	253	67.6%	119	73.5%	134	63.2%	0.036
	Island (Lemnos)	121	32.4%	43	26.5%	78	36.8%	
**Frequency of watching TV**	none	7	2.0%	3	2.0%	4	2.0%	0.812
	1–2 times/week	73	20.7%	32	21.3%	41	20.3%	
	3–4 times/week	89	25.3%	34	22.7%	55	27.2%	
	every day	183	52.0%	81	54.0%	102	50.5%	
**Watching TV (h/d)**	none	8	2.3%	3	2.0%	5	2.5%	0.367
	1 h	190	54.4%	74	49.3%	116	58.3%	
	2–3 h	125	35.8%	60	40.0%	65	32.7%	
	>4 h	26	7.4%	13	8.7%	13	6.5%	
**Screen time (laptop, computer, tablet, mobile phone)**	1–2 h/week	69	19.4%	26	17.0%	43	21.2%	0.067
	3–4 h/week	58	16.3%	18	11.8%	40	19.7%	
	1–2 h/day	138	38.8%	62	40.5%	76	37.4%	
	>3 h/day	91	25.6%	47	30.7%	44	21.7%	
**Video gaming (h/d)**	0–1 h	129	42.3%	37	28.7%	92	52.3%	<0.001 *
	2–3 h	118	38.7%	57	44.2%	61	34.7%	
	4 or more hours	58	19.0%	35	27.1%	23	13.1%	
**Breakfast consumption frequency**	every day	204	71.1%	82	73.2%	122	69.7%	0.693
	1–2 times/week	38	13.2%	15	13.4%	23	13.1%	
	3–5 times/week	45	15.7%	15	13.4%	30	17.1%	
**Beverage consumption**	YES	242	65.8%	113	70.6%	129	62.0%	
	NO	126	34.2%	47	29.4%	79	38.0%	
**Fast food consumption/ week**	none	87	23.5%	34	21.3%	53	25.2%	0.437
	every day	21	5.7%	11	6.9%	10	4.8%	
	1–2 times/week	230	62.2%	104	65.0%	126	60.0%	
	3–4 times/week	32	8.6%	11	6.9%	21	10.0%	
**KIDMED score (0–12)**	poor score < 3	33	12.4%	16	15.0%	17	10.7%	0.307
	medium score 4–7	130	48.9%	55	51.4%	75	47.2%	
	high score > 8	103	38.7%	36	33.6%	67	42.1%	

* *p* < 0.05.

**Table 2:** Data analysis to evaluate the influence of various screen time categories on children’s specific dietary habits.

Variables		Hours/Day in Front of Screens (Laptop, Computer, Tablet, Mobile Phone)								Chi-Square Test of Association				
		1–2 h/week		3–4 h/week		1–2 h/day		>3 h/day						
		Ν	%	Ν	%	Ν	%	Ν	%	*p*-Value				
**Cooking with parents**	YES	46	67.6%	44	77.2%	93	67.9%	51	56%	0.057				
	NO	22	32.4%	13	22.8%	44	32.1%	40	44%					
**Supermarket with parents**	YES	61	91%	51	87.9%	130	94.2%	74	83.1%	0.057				
	NO	6	9%	7	12.1%	8	5.8%	15	16.9%					
**Frequency of taking in school homemade snack**	1–2 times/week	6	9.5%	8	14.8%	12	9.9%	14	18.9%	0.026 *				
	3–5 times/week	7	11.1%	13	24.1%	30	24.8%	23	31.1%					
	every day	50	79.4%	33	61.1%	79	65.3%	37	50%					
**Daily breakfast consumption**	YES	63	92.6%	45	77.6%	110	80.3%	64	71.1%	0.010 *				
	NO	5	7.4%	13	22.4%	27	19.7%	26	28.9%					
**Beverage consumption**	YES	31	47%	37	66.1%	92	67.6%	70	76.9%	0.001 *				
	NO	35	53%	19	33.9%	44	32.4%	21	23.1%					
**Fast food consumption per week**	none		44.8%	15	26.3%	22	16.2%	11	12.1%	0.011 *				
	1–2 times/week	30	44.8%	34	59.6%	98	72.1%	60	65.9%					
	3–4 times/week	4	6%	5	8.8%	9	6.6%	13	14.3%					
	every day	3	4.5%	3	5.3%	7	5.1%	7	7.7%					
**Variables**		**Hours/Day of TV Viewing**								**Chi-Square Test of Association**				
		**0**		**1 h**		**2** **–** **3 h**		**>3 h/day**						
		**Ν**	**%**	**Ν**	**%**	**Ν**	**%**	**Ν**	**%**	***p*** **-Value**				
**Cooking with parents**	YES	2	100%	47	60.3%	40	76.9%	9	56.3%	0.156				
	NO	0	0%	31	39.7%	12	23.1%	7	43.8%					
**Supermarket with parents**	YES	2	66.7%	73	93.6%	47	90.4%	12	75%	0.269				
	NO	1	33.3%	5	6.4%	5	9.6%	4	25%					
**Frequency of taking in school homemade snack**	1–2 times/week	0	0%	12	17.4%	5	10.4%	0	0%	0.560				
	3–5 times/week	0	0%	14	20.3%	12	25.%	3	21.4%					
	every day	2	100%	43	62.3%	31	64.6%	11	78.6%					
**Daily breakfast consumption**	YES	2	66.7%	64	82.1%	41	78.8%	12	75%	0.680				
	NO	1	33.3%	14	17.9%	11	21.2%	4	25.%					
**Beverage consumption**	YES	4	50%	108	58.1%	89	72.4%	23	88.5%	0.030 *				
	NO	4	50%	78	41.9%	34	27.6%	3	11.5%					
**Fast food consumption per week**	none	2	25%	54	29%	21	16.9%	3	11.5%	0.024 *				
	1–2 times/week	2	25%	110	59.1%	84	67.7%	18	69.2%					
	3–4 times/week	2	25%	12	6.5%	14	11.3%	3	11.5%					
	every day	2	25%	10	5.4%	5	4.0%	2	7.7%					
**Variables**		**Hours Video Gaming** **/day**												
		**0**		**1**		**2**		**3**		**4**		**≥5 h**		
		**Ν**	**%**	**Ν**	**%**	**Ν**	**%**	**Ν**	**%**	**Ν**	**%**	**Ν**	**%**	***p*** **-Value**
**Cooking with parents**	YES	37	78.7%	62	76.5%	51	66.2%	24	58.5%	18	60%	14	50%	0.035 *
	NO	10	21.3%	19	23.5%	26	33.8%	17	41.5%	12	40%	14	50%	
**Supermarket with parents**	YES	45	95.7%	74	92.5%	72	93.5%	37	90.2%	26	86.7%	23	82.1%	0.269
	NO	2	4.3%	6	7.5%	5	6.5%	4	9.8%	4	13.3%	5	17.9%	
**Frequency of taking in school homemade snack**	every day	36	76.6%	47	62.7%	33	50.8%	20	58.8%	11	42.3%	18	72%	0.243
	1–2 times/week	1	2.1%	10	13.3%	14	21.5%	3	8.8%	8	30.8%	2	8%	
	3–5 times/week	10	21.3%	18	24%	18	27.7%	11	32.4%	7	26.9%	5	20%	
**Daily breakfast consumption**	YES	38	80.9%	68	84%	59	76.6%	30	73.2%	21	72.4%	18	64.3%	0.680
	NO	9	19.1%	13	16%	18	23.4%	11	26.8%	8	27.6%	10	35.7%	
**Beverage consumption**	YES	25	54.3%	50	62.5%	53	69.7%	29	70.7%	26	86.7%	27	96.4%	<0.001 *
	NO	21	45.7%	30	37.5%	23	30.3%	12	29.3%	4	13.3%	1	3.6%	
**Fast food consumption per week**	none	14	29.8%	18	22.5%	15	19.7%	5	12.5%	3	10%	1	3.6%	0.028 *
	every day	0	0%	3	3.8%	6	7.9%	2	5%	4	13.3%	2	7.1%	
	1–2 times/week	29	61.7%	51	63.7%	49	64.5%	30	75%	17	56.7%	23	82.1%	
	3–4 times/week	4	8.5%	8	10%	6	7.9%	3	7.5%	6	20%	2	7.1%	

* *p* < 0.05.

**Table 3:** Results of One-Way Analysis of Variance (ANOVA) for the association between KIDMED score or Natural Functional Foods consumption and Hours/day in front of screens (laptop, computer, tablet, mobile phone), TV viewing frequency and Hours of video gaming per day.

Variables	Hours/Day in Front of Screens (Laptop, Computer, Tablet, Mobile Phone)	N	ΜO	ΤA	F	*p*-Value
**KIDMED score**	1–2 h/week	66	7.6212	2.42275	7.937	<0.001 *
	3–4 h/week	55	6.7455	2.44357		
	1–2 h/day	133	6.7594	2.38731		
	>3 h/day	82	5.6585	2.63977		
**Natural Functional Foods consumption**	1–2 h/week	69	3.4695	0.7738	7.227	<0.001 *
	3–4 h/week	58	3.368	0.75888		
	1–2 h/day	138	3.1833	0.73704		
	>3 h/day	91	2.9504	0.7612		
	**TV viewing frequency**	N	ΜO	ΤA	F	***p*** **-value**
**KIDMED score**	none	7	5.43	4.35	5.4	0.001 *
	1–2 times/week	72	7.40	2.54		
	3–4 times/week	83	6.92	2.35		
	Every day	172	6.15	2.43		
**Natural Functional Foods consumption**	none	7	3.27	0.84	9.4	<0.001 *
	1–2 times/week	73	3.44	0.78		
	3–4 times/week	89	3.40	0.65		
	Every day	183	3.00	0.76		
	**Hours of video gaming per day**	N	ΜO	ΤA	F	***p*** **-value**
**KIDMED score**	0	47	7.0638	2.6817	2.95	0.013 *
	1	79	7.0253	2.48573		
	2	73	6.6712	2.53889		
	3	36	6.2222	2.36777		
	4	28	5.4286	2.39488		
	5 or more hours	27	5.5926	2.76321		
**Natural Functional Foods consumption**	0	48	3.2804	0.79706	2.75	0.019 *
	1	81	3.3222	0.73073		
	2	77	3.2864	0.79241		
	3	41	3.0342	0.56362		
	4	30	3.0839	0.86578		
	5 or more hours	28	2.8197	0.6679		

* *p* < 0.05.

**Table 4:** Descriptive characteristics of parents, total and by gender.

Variables		Parents						Chi-Square Test of Association *p*-Value
		Total		Mothers		Fathers		
		N	%	N	%	N	%	
**Region**	City (Thessaloniki)	105	66%	92	66.2%	13	65%	0.917
	Island (Lemnos)	54	34%	47	33.8%	7	35%	
**Occupation type**	private employee	54	34%	49	35.3%	5	25%	0.679
	public employee	52	32.7%	44	31.7%	8	40%	
	freelancer/self-employed	31	19.5%	26	18.7%	5	25%	
	Unemployed/Household/other	22	13.8%	20	14.4%	2	10%	
**Education**	Complete High School	29	18.2%	23	16.5%	6	30%	0.333
	Institute of Vocational Training	32	20.1%	29	20.9%	3	15%	
	College-educated	98	61.6%	87	62.6%	11	55%	
**Annual income**	<15,000€	50	31.4%	47	33.8%	3	15%	0.207
	15,000–30,000€	88	55.3%	75	54.0%	13	65%	
	>30,000€	21	13.2%	17	12.2%	4	20%	
**Smoking**	YES	60	37.7%	53	38.1%	7	35%	0.787
	NO	99	62.3%	86	61.9%	13	65%	
**Beverage consumption**	YES	53	33.3%	42	30.2%	11	55%	0.028 *
	NO	106	66.7%	97	69.8%	9	45%	
**Fast food consumption/week**	none	62	39%	57	41%	5	25%	0.612
	1 time/ween	73	45.9%	62	44.6%	11	55%	
	2 times/week	18	11.3%	15	10.8%	3	15%	
	3–5 times/week	2	1.3%	2	1.4%	0	0%	
	every day	4	2.5%	3	2.2%	1	5%	
**Exercise/week**	none	45	28.3%	37	26.6%	8	40%	0.427
	1–2 times/week	82	51.6%	74	53.2%	8	40%	
	>3 times/week	32	20.1%	28	20.1%	4	20%	

* *p* < 0.05.

**Table 5:** Parents’ BMI and MedDiet Score/ children’s BMI total and by gender.

Variables	Gender						*t*-Test *p*-Value
	Total		Women/Girls		Men/Boys		
	M	SD	M	SD	M	SD	
**Parents’ BMI**	24.75	4.70	24.35	4.73	27.67	3.27	0.004 *
**children’s BMI**	18.62	3.12	18.62	3.20	18.60	2.59	0.981
**Parents’ MedDiet Score**	34.04	3.17	34.12	3.05	33.50	3.97	0.508

* *p* < 0.05.

**Table 6:** Pearson correlation coefficients for the association of children’s BMI, parents’ BMI, KIDMED score and MedDiet score.

Variables	Children’s BMI	KIDMED Score
**KIDMED** **score**	−0.190 *	--
**MedDiet** **score**	−0.029	0.144
**Parents’ BMI**	0.314 **	−0.118

* *p* < 0.05. ** *p* < 0.01.

**Table 7:** Family clusters (parent-child).

Variables	Total Sample (N = 137)		Family Clusters (Parent-Child Dyads)			
			1st Cluster—Families with Healthier Eating Habits (n = 70)		2nd Cluster—Families with Less Healthy Eating Habits (n = 67)	
	M	SD	M	SD	M	SD
**Parents’ BMI**	24.68	4.67	22.98_a_	3.73	26.47_b_	4.92
**Children’s BMI**	18.61	3.00	17.51_a_	2.17	19.77_b_	3.31
**MedDiet score**	33.96	3.10	35.49_a_	2.89	32.36_b_	2.44
**Parents’ consumption of modified Functional Foods**	3.04	0.96	3.20_a_	1.03	2.88_b_	0.85
**Parents’ consumption of natural Functional Foods**	3.18	0.53	3.42_a_	0.51	2.92_b_	0.43
**KIDMED score**	6.64	2.45	7.76_a_	2.10	5.46_b_	2.25
**Children’s consumption of natural Functional Foods**	3.19	0.69	3.41_a_	0.71	2.96_b_	0.58
**Number of meals children eat per day**	4.58	1.13	4.89_a_	0.96	4.25_b_	1.21

Note 1. Clusters extracted with the Two Step Cluster Method. 2. Values in the same row not sharing the same subscript are significantly different at *p* < 0.05 in the two-sided independent samples *t*-tests.

**Table 8:** Results of associations between family clusters and various variables.

Variables		Family Clusters				
		Families with Healthier Eating Habits (n = 70)		Families with Less Healthy Eating Habits (n = 67)		Chi-Square Test of Association *p*-Value
		N	%	N	%	
**Parents’ fitness program/week**	none	13	18.6%	22	32.8%	0.068
	1–2 times/week	39	55.7%	36	53.7%	
	>3 times/week	18	25.7%	9	13.4%	
**Watching TV**	YES	68	97.1%	65	97%	0.674
	NO	2	2.9%	2	3%	
**TV viewing frequency**	none	1	1.5%	2	3%	0.708
	1–2 times/week	18	26.5%	13	19.4%	
	3–4 times/week	15	22.1%	14	20.9%	
	every day	34	50%	38	56.7%	
**TV viewing hours**	none	1	1.5%	2	3.1%	0.197
	1 h	40	59.7%	28	43.1%	
	2–3 h	22	32.8%	26	40%	
	>4 h	4	6%	9	13.8%	
**Hours/day in front of screens (laptop, PC, tablet, mobile phone)**	none	0	0%	0	0%	0.065
	1–2 h/week	17	24.6%	8	12.7%	
	3–4 h/week	15	21.7%	16	25.4%	
	1–2 h/day	29	42.0%	22	34.9%	
	>3 h/day	8	11.6%	17	27.0%	
**Supermarket with parents**	YES	64	91.4%	59	88.1%	0.515
	NO	6	8.6%	8	11.9%	
**Cooking with parents**	YES	51	72.9%	42	63.6%	0.273
	NO	19	27.1%	24	36.4%	
**Videogaming hours/day**	0 h	11	19%	9	15.8%	0.720
	1 h	18	31%	17	29.8%	
	2 h	17	29.3%	12	21.1%	
	3 h	6	10.3%	8	14%	
	4 h	3	5.2%	5	8.8%	
	≥5 h	3	5.2%	6	10.5%	
**Frequency of Breakfast consumption in children**	every day	45	75%	37	74%	0.758
	1–2 times/week	6	10%	7	14%	
	3–5 times/week	9	15%	6	12%	
**Frequency of Fast Food consumption in parents**	none	30	42.9%	25	37.3%	0.802
	1–2 times/week	38	54.3%	40	59.7%	
	≥3 times/week	2	2.9%	2	3%	
**Frequency of Fast-Food consumption in children**	none	21	30%	19	28.4%	0.049 *
	1–2 times/week	45	64.3%	35	52.2%	
	≥3 times/week	4	5.7%	13	19.4%	
**Children’s Beverage consumption**	YES	43	63.2%	46	68.7%	0.506
	NO	25	36.8%	21	31.3%	
**Sweet consumption/week**	none	6	8.6%	1	1.5%	0.231
	every day	13	18.6%	17	25.4%	
	1–2 times/week	31	44.3%	32	47.8%	
	3–4 times/week	20	28.6%	17	25.4%	

* *p* < 0.05.

## Data Availability

The data presented in this study are available upon request from the corresponding author.
